# Age-related changes in the gene expression profile of antigen-specific mouse CD8+ T cells can be partially reversed by blockade of the BTLA/CD160 pathways during vaccination

**DOI:** 10.18632/aging.101105

**Published:** 2016-11-09

**Authors:** Noor Dawany, Elizabeth M Parzych, Louise C Showe, Hildegund CJ Ertl

**Affiliations:** ^1^ Wistar Institute, Philadelphia, PA 19104, USA; ^2^ Present Address: The Children's Hospital of Philadelphia, Department of Biomedical and Health Informatics, Philadelphia, PA 19104, USA; ^3^ Present Address: Drexel University, School of Medicine, Philadelphia, PA 19104, USA

**Keywords:** gene expression, vaccination, aging, BTLA/CD160, CD8+ cells

## Abstract

We analyzed gene expression profiles of young and aged mouse CD8^+^ T cells specific for the nucleoprotein (NP) of influenza A/PR8/34 virus. CD8^+^ T cells were stimulated either by the NP antigen expressed in its native form or fused into the herpes virus (HSV)-1 glycoprotein D (gD) protein, which blocks signaling through the immunoinhibitory B and T lymphocyte attenuator (BTLA) and CD160 pathways. We show that NP-specific CD8^+^ T cells from aged mice exhibit numerous differences in gene expression compared to NP-specific CD8^+^ T cells from young mice, including a significant reduction of expression in genes involved in T cell receptor (TcR) and CD28 signaling. We also show that these changes can be reversed in a sub-population (∼50%) of the aged mice by a BTLA/CD160 checkpoint blockade. These results suggest that BTLA/CD160 checkpoint blockade has potential value as a vaccine additive to induce better CD8^+^ T cell responses in the aged.

## INTRODUCTION

It is well documented that immune responses decline with age, leaving the elderly less responsive to vaccines and increasingly susceptible to infections and cancer [1-4]. We previously demonstrated both intrinsic and extrinsic defects in CD8^+^ T cells of aged mice [5]. Aged mice are deficient in naïve CD8^+^ T cells and show a paucity of specific T cell precursors [5]. Aged T cells also increase expression of co-inhibitory markers that are typically associated with T cell exhaustion [6,7]. We also showed that blockade of the co-inhibitors BTLA/CD160 increases antigen-driven CD8^+^ T effector cell responses, especially in aged mice [8]. To assess the basis for these defects in CD8^+^ T cells from the aged and determine how they are rescued by BTLA/CD160 checkpoint blockade, we compared the global transcriptomes of CD8^+^ T cells from young and aged mice that had been immunized with E1-deleted adenovirus (Ad) vectors expressing the influenza A/PR8/34 virus NP. NP was either fused into HSV-1 gDor in its native form or with HSV-1 gD, which we have shown inhibits BTLA and CD160's binding to their ligand, the Herpes Virus Entry Mediator (HVEM) [8-10], and increases vaccine-driven CD8^+^ T cell responses in young and aged mice.

Co-stimulatory and co-inhibitory molecules on the surface of T cells that interact with corresponding ligands on antigen presenting cells (APCs) tightly control CD8^+^ T cell responses [11,12]. CD28, the major co-stimulator on CD8^+^ T cells, binds to CD80 or CD86 on antigen-presenting cells (APCs). Ligation of the T cell receptor (TcR) to the MHC I – peptide complex results in activation of the nuclear factor of kappa light polypeptide gene enhancer in B-cells (NF-κB), c-Jun/c-Fos, rat Sarcoma viral oncogene homolog (Ras) and nuclear factor of activated T cells (NFAT)c pathways, which regulate transcription, apoptosis and induce translation of key effector molecules, such as cytokines and other molecules that affect cell motility and homing patterns [13]. CD28 signaling, which is essential for full activation of T cells through increased activation of lymphocyte-specific protein tyrosine kinase (Lck), amplifies TcR signaling [14]. Furthermore, signaling through CD28 enhances activation of the phosphatidylinositol-4,5-bisphosphate 3-kinase (PI3K) complex [15]. This complex phospho-rylates pyruvate dehydrogenase kinase (PDK)1, which in turn stimulates protein kinase B (AKT). Signaling through AKT, in part, increases the effect of TcR signaling by enhancing activation of the NF-kB and Ras pathways [16]. AKT signaling induces protein synthesis through the activation of mammalian target of rapamycin (mTOR) [17]. Furthermore, AKT, in collaboration with TcR signaling, increases glucose uptake as well as total cellular and mitochondrial hexokinase activity, thus accommodating the metabolic needs of rapidly proliferating cells by promoting glycolysis [18].

Co-inhibitory molecules such as cytotoxic T-lymphocyte-associated protein 4 (CTLA-4) or programmed cell death protein (PD-)1, which are both expressed after T cell stimulation, counterbalance T cell activation through TcR and CD28 ligation. Specifically, CTLA-4, which has 10-100 times higher affinity for CD80/86 than CD28, activates tyrosine phosphatase (SHP)-2 and protein phosphatase (PP)2A, which then dephosphorylate downstream molecules of the TcR signaling cascade. PP2A specifically blocks AKT activation downstream of PI3K. PD-1 also inhibits AKT through blockade of PI3K activation upon recruitment of SHP-1 and SHP-2 to the immunoinhibitory tyrosine motif in its cytoplasmic tail [19].

BTLA is another immunoinhibitory molecule that, unlike CTLA-4 or PD1, is constitutively expressed on naïve T cells and thereby likely affects the very early steps of T cell activation. BTLA on T cells interacts with HVEM on APCs [20]. HVEM, in addition, binds to CD160, an immunoinhibitory molecule that is induced upon T cell activation [21]. It also interacts with two co-stimulators, i.e., tumor necrosis factor superfamily member 14 (LIGHT) and lymphotoxin alpha (LTɑ), as well as with gD of HSV-1 [22]. BTLA, CD160 and HSV gD bind to the cysteine-rich domain (CRD) 1 of HVEM while LIGHT and LTɑ bind to CRD2/3. HSV gD blocks HVEM binding of CD160 and BTLA but neither of these three molecules interferes with the binding of LIGHT or LTɑ. Upon binding of both inhibitory and stimulatory molecules, signaling through the inhibitors is dominant. HSV gD, by blocking binding of BTLA/CD160 to HVEM, allows for signaling through LIGHT and/or LTɑ [23].

Here we report on an analysis of the transcriptomes of mouse effector CD8^+^ T cells, specific to the NP of influenza A virus, that were stimulated by vaccines. We compare NP-specific CD8^+^ T cells from young and aged mice that were immunized with either a native form of NP or a fusion protein of HSV-1 gD and NP (gDNP), designed to block the BTLA/CD160 pathways during CD8^+^ T cell stimulation. Our results show numerous differences between the aged and young CD8^+^ T cells. Many of these changes appear to be linked to the downregulation of the CD28/TcR signaling pathways, confirming previous results [24]. Blockade of BTLA/CD160 signaling during stimulation of aged T cells reverses the changes in CD28/TcR signaling in half of the mice where the global transcriptomic profile becomes similar to what is typically observed in young mice. This suggests a potential role for gD as an adjuvant for vaccines designed to induce CD8^+^ T cells in a significant subset of the aged.

## RESULTS

### Experimental design and overall results

Young (n=5) and aged (n=5) female C57Bl/6 mice were immunized with an E1-deleted adenovirus vector derived from chimpanzee virus SAd-V-25 (also termed AdC68) expressing the NP of A/PR8/34 influenza virus in its native form (AdC68-NP). Additional young (n=7) and aged (n=10) mice were vaccinated with NP fused into gD of the HSV-1 (AdC68-gDNP). NP-specific CD8^+^ T cells were isolated from the spleen at the approximate height of the response, 14 days for young mice or 20 days for aged mice, by cell sorting with staining for live cells, CD8 and an APC-labeled NP tetramer for the immunodominant H-2^b^ class I binding epitope of NP (amino acid sequences 366-374). RNA was extracted and then analyzed for global gene expression on the Illumina MouseWG-6 v2 gene expression microarrays.

**Figure 1:**
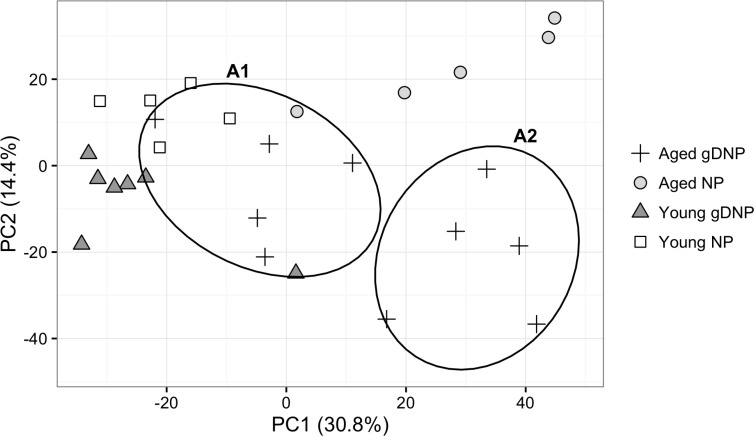
Dimensional reduction of the 2,362 significant probes identified using ANOVA at p<0.05 The first 2 principal components account for 45% of the variation in the data. Samples from Aged AdC68-gDNP mice cluster into two groups (A1 and A2).

Gene expression analyses were carried to compare the response of both age groups with one vaccine treatment or in one age group with both vaccine treatments. We identified 2,362 genes differentially expressed at p<0.05 between the 4 classes. The unsupervised principal component analysis (PCA) based on these genes shows that samples from young mice cluster by vaccine type, but the differences are relatively small (Fig. 1). Samples from aged AdC68-NP-immunized mice form a distinct cluster with the AdC68-gDNP-immunized mice being roughly divided into two clusters: one (A1) that co-localizes with the samples from young AdC68-gDNP-immunized mice and another (A2) that is clearly distinct from the other groups.

### Comparing samples from young mice of the two different vaccines groups

In young mice, differences between the two vaccine groups were small and some of the mice showed similar or overlapping patterns. Only 94 genes were identified as significantly differentially expressed between NP-specific CD8^+^ T cells from young mice that received the vaccine with or without gD, and none of them showed differences in expression ≥1.5-fold change (FC). Of interest was the differential expression of nuclear factor for activated T cells (*Nfat*)*c1*, which facilitates expression of cytokine genes such as IL-2 and IL-4; this gene was expressed higher (1.4 fold) in CD8^+^ T cells from AdC68-gDNP immunized young mice. Lymphoid enhancing binding factor 1 (*Lef1*), which regulates T-cell receptor alpha enhancer function, in contrast was under-expressed in the same comparison (−1.3 fold) (Suppl. Fig. 1A). Ingenuity pathway analysis showed enrichment of the SAPK/JNK signaling (p=5.9×10^−4^, 4 genes) and the Wnt/β-catenin signaling (p=6.2×10^−4^, 5 genes) pathways (Suppl. Table 1). None of the pathways or functions analyzed by DAVID reached statistical significance.

### Comparing samples from aged mice of the two different vaccines groups

NP-specific CD8^+^ T cells from aged mice in general were more variable. There were 412 differentially expressed transcripts when comparing NP-specific CD8^+^ T cells from aged mice that received the AdC68-gDNP vaccine and those from AdC68-NP-vaccinated aged mice, 105 of which had an FC≥1.5. Transcripts encoding proteins involved in metabolism, antigen presentation or inflammatory responses were largely expressed at lower levels in gDNP-induced CD8^+^ T cells, while those involved in T cell activation such as mTOR (*Frap1*) or *Nfat5* were higher (Suppl. Fig. 1B). Several pathways in Ingenuity, such as the Inducible T-cell co-stimulator (ICOS)-ICOS-L signaling in T helper cells (p=1.9×10^−3^, 7 genes), were significantly different between the two groups (Suppl. Table 2). We selected 6 genes (Klrg1, Lyz, Cd163, Sirpa, Pik3cb, Aif1, Lat2) for validation using qRT-PCR. All 6 genes displayed the same directional fold change as the microarray expression data, however only Sirpa reached statistical significance (Supplementary Methods and Results).

### Comparing the two age groups vaccinated with AdC68-NP

Focusing first on NP-specific CD8^+^ T cells isolated from mice immunized with AdC68-NP we see that, in general, samples from aged mice are more variable in their response to the vaccine than those from the young mice and form a less cohesive group in the PCA plot (Fig. 1). The largest differences in gene expression were seen between the young and the aged CD8^+^ T cells induced by the AdC68-NP vaccine as seen in Fig. 1. A total of 474 probes, of which 161 had a FC≥1.5, distinguished T cells from aged and young mice that received the AdC68-NP vaccine (Table 1). As shown in the PCA (Fig. 1), the aged NP-induced CD8^+^ T cells clustered separately from those of AdC68-NP-immunized young mice. Of the 161 probes that were differentially expressed at FC≥1.5, a total of 96 probes showed higher expression in young NP-induced CD8^+^ T cells compared to the 65 probes that where more highly expressed in the aged cells (Table 1).

**Table 1:** Number of differentially expressed probes between the different mice groups

		All Genes		Upregulated†		Downregulated‡	
Group 1	Group 2	All	FC > 1.5	All	FC > 1.5	All	FC > 1.5
Aged AdC68gDNP	Aged AdC6DNP	412	105	100	10	312	95
Young AdC68gDNP	Young AdC68NP	94	0	30	0	64	0
Aged AdC68NP	Young AdC68NP	474	161	184	65	290	96
Aged AdC68gDNP	Young AdC68gDNP	507	90	244	53	263	37
A1	A2	650	253	504	218	146	35
A1	Young AdC68gDNP	113	5	76	4	37	1
A2	Young AdC68gDNP	2257	961	922	271	1335	690
A1	Aged AdC68NP	508	169	369	117	139	52
A2	Aged AdC68NP	835	256	439	86	396	170

† Upregulated in Group1 compared to Group2

‡ Downregulated in Group1 compared to Group2

The expression of metabolic genes was largely reduced in aged CD8^+^ T cells. The pattern of reduced expression of phosphofructokinase (*Pfkb*), a key regulator in glycolysis, and increased expression in peroxisome proliferator activated receptor gamma (*Pparγ*), a transcription factor that activates peroxisomal fatty acid beta oxidization, suggests differences in preferential catabolism of nutrients (Suppl. Fig. 3). Transcripts involved in T cell activation and functions were also largely lower in aged CD8^+^ T cells and these included crucial regulators of activation such as *Lat*, lymphocyte cytosolic protein (*Lcp*)*-1*, *Lef1*, or the *Lck* proto-oncogene (Suppl. Fig. 1C). Some differences were noted in transcripts encoding proteins involved in antigen presentation and processing or inflammatory responses without showing a clear trend toward higher or lower expression.

Pathway analysis in Ingenuity showed a significant reduction in components of the CD28 pathway as well as the related protein kinase PKCθ pathways (Suppl. Table 3). CD28 ligation enhances signaling downstream from the TcR resulting in activation of the NF-κB and mitogen-activated kinase 8 (JNK) pathways. Expression of genes in this part of the CD28 cascade was decreased in aged CD8^+^ T cells. CD28 signaling also activates PI3K, which in turn enhances AKT/mTOR signaling, leading to increased cell metabolism and protein synthesis. It also increases energy production through glycolysis, which provides biomass for proliferating cells. AKT blocks the activity of forkhead box (*Fox*)*O1*, which was moderately decreased in the aged T cells (FC=-1.2). Despite the reduced expression of transcripts encoding proteins involved in TcR/CD28 signaling, *PIK3cb* (FC=1.9), a component of the PI3K complex was significantly increased in the aged cells, while message levels for components for the downstream AKT/mTOR pathways were comparable in young and aged cells, potentially suggesting alternative CD28-independent pathways for activation of this pathway. Another pathway that affects AKT/mTor signaling is initiated by insulin receptor signaling, which showed no significant differences in mRNA expression between young and aged T cells.

Comparing gene functions that were significantly altered between young and aged AdC68-NP-immunized mice we found a strong reduction of functions associated with gene expression, RNA post-transcrip-tional regulation, cell cycle, and cell death, among others (Suppl. Table 3).

### Comparing the two age groups vaccinated with AdC68-gDNP

NP-specific CD8^+^ T cells from AdC68-gDNP-immune aged mice could be distinguished from those of young mice that received the same vaccine by 507 gene probes of which 90 differed by ≥1.5 fold. The PCA showed that aged AdC68-gDNP induced CD8^+^ T cells formed two distinct clusters that differed in 650 gene probes with 253 showing differences ≥1.5 fold change. One cluster, from here on referred to as cluster A1, clustered close to samples from the AdC68-gDNP induced CD8^+^ T cells from young mice. The second cluster, A2, was distinct from all the other groups (Fig. 1).

Expression of genes involved in metabolism tended to be overall lower in aged samples. Levels of transcripts involved in T cell activation and function were largely similar but of note was a significant decrease in *Nfatc1* (FC=-1.6) in aged as compared to young AdC68-gDNP-immunized mice, while *Lat* (FC=2.2) was significantly increased. Nfatc1 is downstream of several important pathways including those initiated by TcR/CD28 signaling. Meanwhile, differences in transcripts encoding proteins involved in antigen processing, presentation or autophagy as well as those participating in inflammatory reactions were largely similar (Suppl. Fig. 1D). Pathway and functional analyses failed to reveal any significant pathway differences between young and aged NP-specific CD8^+^ T cells from AdC68-gDNP immunized mice.

### Comparing the A1 and A2 groups of aged AdC68-gDNP induced NP-specific CD8^+^ T cells

**Figure 2:**
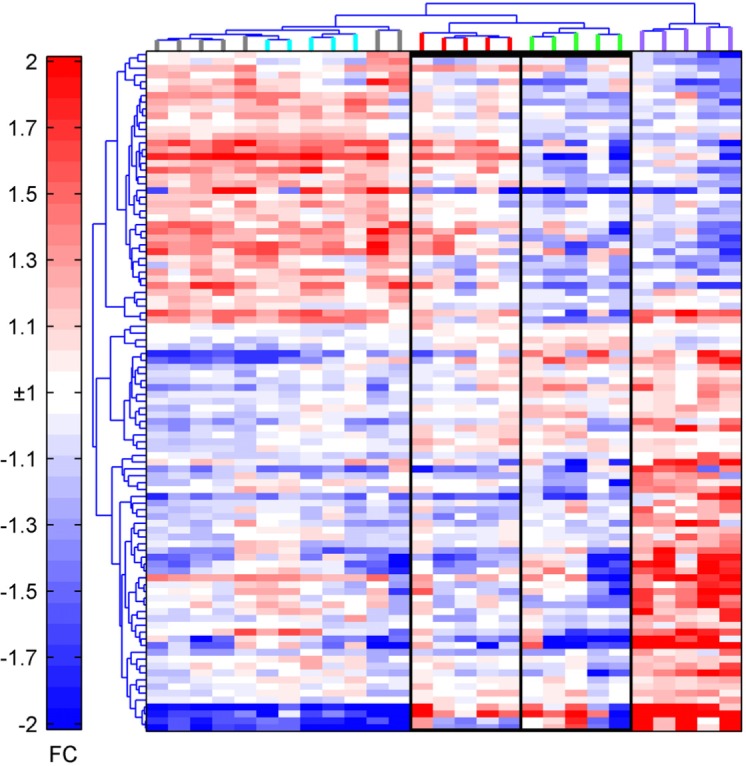
Heatmap of the top 100 probes identified by ANOVA The top probes ranked by p-value clearly separate the samples into two arms, with aged AdC68-gDNP mice clustering into two groups and aged AdC68-NP mice separately from all the other groups. Young AdC68-NP: blue; Young AdC68-gDNP: gray; Aged AdC68-NP: purple; A1: red; A2: green.

We next compared the NP-specific CD8^+^ T cells from the two distinct clusters of AdC68-gDNP-immunzed aged mice. We eliminated the possibility that the separation of the 2 groups was due to an experimental batch effect as members of each group came from several experiments. We also eliminated a technical batch effect of the array processing, as samples from both groups were included in the different array batches. To further understand the nature of this difference we analyzed the array data differently. We first applied unsupervised hierarchical clustering to all the samples using only the top 100 genes, ranked by p value from the ANOVA comparison between the four mice groups (Suppl. Table 4) rather than all the p<0.05 genes probes (2,362) used for the PCA in Fig. 1. Using only 100 genes we see the same separation by clustering of the sample classes in the heatmap (Fig. 2) as was seen by the PCA plot. The samples separate into 2 major clusters indicated by the treeview at the top of the heatmap. The 5 samples from the aged AdC68-NP-induced CD8^+^ T cells cluster at the far right (purple) and are clearly separated from the remaining samples in the large cluster to the left. The left hand cluster separates into 3 branches with all 12 of the young samples in the branch at the far left indicating their relatedness to each other. Although all the samples from the aged AdC68-gDNP-induced CD8^+^ T cells are also in the major cluster with the young CD8^+^ T cell samples, they still separate into the same 2 groups (A1and A2) identified in the PCA analysis (Fig. 1). This indicates that, overall, based on their expression profiles, the aged AdC68-gDNP-induced CD8^+^ T cells are more similar to both classes of young mice than they are to the aged NP-induced CD8^+^ T cells. However the AdC68-gDNP immunized aged mice still form 2 sub-clusters with aged A1 (red) cells being more similar to the young cells and A2 (green) less similar to the young cells. Immunization of mice with the AdC68-gDNP vaccine thus appears to rescue the age-related defects in activated CD8^+^ T cells evident in the aged/young comparisons between AdC68-NP immunized mice, resulting in gene expression profiles that are similar, but not identical, to those typically seen in activated vaccine-induced CD8^+^ T cells from young mice. Furthermore 50% of the aged mice (A1) were more closely related to the young than the A2 mice.

To identify gene signatures that best characterize the A1/A2 gene expression differences, we compared the expression between A1 and A2 of the 2,362 probes using a t-test. We identified 650 significant (p<0.05) genes to be differentially expressed in a comparison between the 2 aged subsets. The majority of those genes (504 in total, including 218 with FC≥1.5) are expressed at higher levels in A1 CD8^+^ T cells as compared to A2 CD8^+^ T cells. Among the largest changes were key molecules in T cell activation and differentiation such as T-bet (encoded by *Tbx21*, FC=1.8), eomesodermin (*Eomes*, FC=2.1), protein tyrosine phosphatase, receptor type C (*Ptprc*, FC=2.3), an essential regulator of TcR signaling and *Fyn* (FC=1.9), which is activated upon TcR signaling. Additional transcripts encoding metabolic enzymes were similarly increased, including phospho-fructokinase, platelet (*Pfkp*, FC=1.7) and succinate dehydrogenase complex subunit C (*Sdhc*, FC=1.9), with the former participating in glycolysis and the latter in the TCA cycle. A number of mitochondrial ribosomal proteins (*Mrps9*, FC=1.5, *Mrps30*, FC=1.4 and *Mrps33*, FC=1.6) as well as participants in the mitochondrial respiratory chain, i.e., NADH:ubiquinone oxidoreduc-tases (*Nduf*)*a1* (FC=2.2), *4* (FC=1.5) *9* (FC=1.5) and *Ndufb6* (FC=1.7) and transcripts for ribosomal proteins (*Rps*)*11* (FC=1.8), *6* (FC=1.4) and *7* (FC=1.6) were also higher in A1 than A2 samples.

### Comparison of aged AdC68-gDNP-induced CD8^+^ T cells of the two clusters with those from AdC68-gDNP-immunized young mice

We compared samples from the A1 and A2 groups of aged AdC68-gDNP-vaccinated mice to samples from young mice vaccinated with AdC68-gDNP. In the gDNP-specific CD8^+^ T cells from the A1 group, where expression patterns were more closely related to those of CD8^+^ T cells from young AdC68-gDNP-immunized mice, 113 genes were differentially expressed between the two groups, 76 of which were upregulated in A1. Limiting the analysis to genes with a FC≥1.5 reduced these numbers to a total of 5 genes, of which 4 were upregulated in A1. Differences were far higher for the A2 group with 2,257 probes reaching statistical significance, of which 922 were expressed higher in A2. Of these 961 reached a FC≥1.5 with 271 expressed higher in A2 (Table 1). Differences in expression of genes involved in metabolism were mainly seen for the A2 group and only 2 of the 23 transcripts showed a similar expression pattern in the A1 group. Transcripts involved in T cell activation were largely reduced in comparison to those from young AdC68-gDNP samples in the A2 group, but increased in the A1 group. Expression of genes involved in antigen processing or presentation were reduced in the A2 group while some were increased in the A1 group. This pattern was less pronounced for transcripts indicative of inflammatory response. Only very few of the transcripts highlighted in Suppl. Fig. 2 showed the same trend in the A1 and A2 groups.

Comparing A1 CD8^+^ T cells to those from AdC68-gDNP immunized young mice showed no significant differences in pathways in Ingenuity. Of the 5 functions in Ingenuity that were statistically significant, none were related to immune responses, and none of the functions analyzed by DAVID were enriched (Suppl. Table 5).

Comparing A2 cells to those from young AdC68-gDNP-immunized mice showed differences in 39 pathways by Ingenuity, with mitochondrial dysfunctions (Suppl. Fig. 3) being the most significant (p=1.7E-0.6, 32 genes). Other pathways that were significant included mTOR signaling (p=3.1×10^−5^, 31 genes), TcR signaling (p=1.1×10^−4^, 19 genes), oxidative phosphorylation (p=1.9×10^−4^, 20 genes), CD28 signaling of T helper cells (Suppl. Fig 4) (p=5.6×10^−4^, 20 genes) and cytotoxic T cell-mediated apoptosis of target cells (p=2.2×10^−3^, 8 genes) (Suppl. Table 6). Most of the genes involved in these pathways were downregulated in A2 cells. Analysis in Ingenuity showed differences in 56 functions including cell-mediated immune responses (p=3.2×10^−8^, 115 genes), protein synthesis (p=5.6×10^−6^, 224 genes), immune cell trafficking (p=8.9×10^−6^, 179 genes), inflammatory responses (p=8.9×10^−6^, 175 genes), carbohydrate metabolism (p=4.7×10^−5^, 5 genes), protein degradation (p=1.8×10^−4^, 91 genes) and antigen presentation (p=4.9×10^−4^, 8 genes). Four KEGG pathways from DAVID were statistically significant, with ribosomes being the most significant (p=3.6×10^−8^, 34 genes). In addition, 11 other functions in DAVID were significant; 8 of which reflected functions of ribosomes or ribonucleoproteins (Suppl. Table 6).

### Comparison of aged AdC68-gDNP-induced CD8^+^ T cells of the A1 and A2 clusters with those from AdC68-NP-immunized aged mice

The comparison of the A1 and A2 group samples to those from aged AdC68-NP-immune samples showed differences in 508 and 835 genes, respectively, of which 169 and 256 reached a FC≥1.5. In the A1 group 369 (117 FC≥1.5) genes were upregulated while in the A2 group 256 (86 FC≥1.5) genes showed higher expression. Looking at individual genes involved in metabolism, T cell activation/functions, antigen presentation and processing or inflammatory reactions showed again that the two groups displayed very distinct differences compared to the samples from aged AdC68-NP-immunized mice. The A1 group showed some differences in expression of genes involved in metabolism and T cell activation or functions and in both cases genes tended to be upregulated. The A2 group showed very pronounced differences in expression levels of genes involved in metabolism and strong reductions in genes encoding ribosomal proteins or proteins involved in antigen presentation or processing. There were also marked differences in genes indicative of inflammatory responses (Suppl. Fig. 5).

Ingenuity pathway analysis showed significant differences between A1 CD8^+^ T cells and those from aged AdC68-NP immunized mice in CD28 signaling in T helper cells (Suppl. Fig. 6) (p=7.8×10^−5^, 10 genes) and PKCθ (p=7.8×10^−5^, 10 genes). A total of 66 functions were also significantly enriched, including immune cell trafficking (p=2.3×10^−6^, 60 genes), inflammatory responses (p=2.3×10^−6^, 80 genes), cell-mediated immune responses (p=1.2×10^−5^, 43 genes) and metabolic diseases (p=3.9×10^−5^, 43 genes). In DAVID, 6 other functions were significant, including positive regulation of immune system process (p=2.8×10^−5^, 17 genes), activation of immune response (p=2.9×10^−5^, 11 genes), immune response-activating cell surface receptor signaling pathway (p=5.0×10^−5^, 8 genes) and immune response-activating signal transduction (p=1.4×10^−4^, 8 genes) (Suppl. Table 7).

None of the Ingenuity pathways reached statistical significance when considering genes differentially expressed between the A2 group and samples from the aged AdC68NP-immunized mice, although there were 31 significant functions including protein synthesis (p=4.8×10^−7^, 76 genes), protein degradation (p=4.8×10^−7^, 49 genes), immune cell trafficking (p=7.8×10^−5^, 79 genes) and inflammatory responses (p=7.8×10^−5^, 98 genes). Analysis by DAVID showed no significantly enriched pathways or functions (Suppl. Table 8).

### Confirmatory studies

We analyzed the numbers of CD8^+^ T cells and tetramer^+^CD8^+^ T cells that were recovered from each sample to determine if absolute numbers of antigen-specific CD8^+^ T cells were similar between clusters. Comparing results for the A1 and A2 samples of the aged AdC68-gDNP-immunized mice showed high variability in each group but no significant differences between samples of the two groups (Suppl. Fig. 7).

We conducted an analysis for expression of the TcR by determining intensity of the tetramer stain combined with intensity of the stain for CD28 using blood samples from additional young and aged mice vaccinated with the two vaccines. We assessed activated (CD44^+^) tetramer^+^CD8^+^ at 2 and 4 weeks after immunization and naïve (CD44^−^) CD8^+^ T cells for expression of the latter at 2 weeks after vaccination. As shown in Suppl. Fig. 8, there were no significant differences in levels of TcR expression between young and aged CD8^+^ T cells. CD28, in contrast to previous reports testing activated mouse T cells [25], was significantly higher on naïve CD8^+^ T cells from aged mice; on NP-specific CD8^+^ T cells expression levels were very variable but failed to be significantly different from those observed on young CD8^+^ T cells.

## DISCUSSION

Here we report on results from an analysis of gene expression profiles of splenic mouse CD8^+^ T cells. Unlike in previous studies that reported on gene expression in human or mouse T cells [26-28], we analyzed highly purified antigen-specific CD8^+^ T cells that had been induced by a vaccine to the NP of influenza virus. We observed a reduction in expression of genes involved in transcription, biosynthesis and metabolism in CD8^+^ T cells from aged AdC68-NP-vaccinated mice. Our analysis also showed a reduction in TcR/CD28 signaling, confirming previous reports [29-31].

Levels of cell surface expressed NP epitope-specific TcR were slightly lower in aged CD8^+^ T cells although this difference failed to reach significance. Measurements of CD28 levels showed increased expression on aged naïve CD8^+^ T cells. Levels of CD28 in activated aged NP-specific CD8^+^ T cells were highly variable but not significantly different from those of younger mice. These results, which contrast previous reports using unsorted CD8+ T cells from mice or humans [25], indicated that a mere reduction in expression of the TcR or CD28 could not explain the overall reduction of other mediators of these pathways.

Our main emphasis in this study was on defining age-related changes in antigen-activated CD8^+^ effector T cells that could be reversed when BTLA/CD160 signaling was blocked during activation. As we reported previously [8], aged mice immunized with an Ad vector expressing the NP of influenza A virus in comparison to young mice develop reduced NP-specific CD8^+^ T cell responses, which can be reversed by expressing the antigen within HSV-1 gD. The gD fusion protein also increases CD8^+^ T cell-mediated cytolysis while production of cytokines remains unaffected. Using unrelated tumor-associated antigens such as E7 of human papilloma virus (HPV)-16 or a series of melanoma-associated epitopes in tumor-bearing young mice, we confirmed that gD-mediated BTLA/CD160 blockade increases CD8^+^ T cell responses, especially to subdominant epitopes, renders CD8^+^ T cells less susceptible to exhaustion within a tumor micro-environment and improves the T cells’ ability to reduce tumor progression [9,10].

Data presented here show that transcriptional differences, upon inhibiting BTLA/CD160 signaling by gD, were more pronounced in aged than young CD8^+^ T cells. Interestingly, the age-related defects were only reversed by blockade of this pathway in NP-specific CD8^+^ T cells from half of the aged mice (5 out of 10) referred to as the A1 group. The CD8^+^ T cell gene expression profile of the other aged mice, although it remained distinct from that of CD8^+^ T cells from AdC68-NP-vaccinated aged mice, continued to show marked differences to those from younger mice. An analysis of numbers or frequencies of CD8^+^ T cells or NP-specific CD8^+^ T cells conducted during cell sorting failed to predict which of the AdC68-gDNP-immunzed mice would cluster within A1 or A2.

In younger mice inclusion of gD into the vaccine had only minor effects on the CD8^+^ T cells’ transcriptome during the height of the effector phase. However, as reported previously [9], the addition of gD increased the overall magnitude of NP-specific CD8^+^ T cell responses. In the aged A1 group inclusion of gD reversed the defects in CD28 and TcR signaling and related downstream pathways such as mTOR signaling, which in turn increased transcripts encoding factors involved in protein synthesis, cell survival and metabolism, when compared to aged AdC68-NP-induced CD8^+^ T cells. In contrast the aged A2 group tended to show significantly lower expression of transcripts encoding key proteins of T cell activation, protein synthesis or metabolism compared to CD8^+^ T cells from young AdC68-gDNP-immunized aged mice.

Especially striking were the differences in transcripts encoding metabolic proteins in the comparisons of the A1 and A2 groups. After recognition of their cognate antigen through the TcR on APCs that provide costimulation through CD28, CD8^+^ T cells initially undergo metabolic switches. While naïve T cells gain energy primarily through the highly efficient TCA cycle, they switch, upon activation of the AKT/mTOR and hypoxia induced factor (HIF)-1ɑ pathways, to glycolysis. Glycolysis is less efficient in generating energy through ATP, but provides crucial building blocks for dividing cells [32]. Samples of the A1 group, in comparison to those from aged AdC68-NP-immunized mice, showed a 1.9 fold increase in phosphofructokinase, the first enzyme that commits glucose to catabolism through glycolysis by phosphorylating D-fructose 6-phosphate to fructose 1,6-bisphosphate, suggesting that A1 cells were better equipped to undergo metabolic reprogramming to this pathway. Samples from the A2 group showed a reduction in succinate dehydrogenase, which is part of a key enzyme complex of the TCA cycle and the aerobic respiratory chain of mitochondria, in comparison to samples from young AdC68-gDNP immunized mice. This, combined with reductions in other subunits of the electron transport chain and additional mitochondrial proteins including fission, mitochondrial 1 (*Fis1*), which mediates mitochondria fission, suggests that mitochondrial dysfunctions, which are known to afflict aged cells [33], are reversed in the A1 group. Further reductions in the glycolytic enzymes phospho-fructokinase and pyruvate kinase combined with increases in pyruvate dehydrogenase kinase 4 (*Pdk4*), which inhibits the pyruvate dehydrogenase complex and thereby the conversion of pyruvate to acetyl-coenzyme A in A2 cells, indicate reduced energy production through glycolysis. Overall these data suggest that, in the aged CD8^+^ T cells from the A2 but not the A1 group, energy production through both aerobic glycolysis and the mitochondrial TCA cycle declined compared to the younger AdC68-gDNP-immunized mice. This decrease in energy production would be expected to impact the cells’ ability to proliferate, function and survive.

How could age-related defects be adjusted by HSV-gD-mediated blockade of the BTLA/CD160-HVEM pathways? Signaling through HVEM is complex. HVEM interacts with LIGHT and LTɑ, two costimulatory molecules, and concomitantly with BTLA or CD160. Mice lacking BTLA or HVEM show increased susceptibility to autoimmune diseases [34,35] but improved immune responses to some infectious agents [36] confirming that BTLA and HVEM have immunoinhibitory functions. Binding of BTLA or CD160 to HVEM is blocked by gD. Although the downstream signaling events of these different molecules upon binding to HVEM are not yet fully understood, available evidence indicates that LIGHT and LTɑ activate the NF-kB pathway through TNF receptor-associated factor (TRAF)-2 [37]. BTLA contains three cytoplasmic tyrosine based immuno-inhibitory motifs, which recruit SHP-1 and SHP-2 [38]. SHP-1 provides a negative feedback to Lck, which is activated upon TcR ligation. This activation is sustained by CD28 signaling. Lck phosphorylates tyrosines residues within the immunoreceptor tyrosine-based activation motifs (ITAM) of the cytoplasmic tails of the TcR-gamma chains and CD3 subunits, initiating the TcR/CD3 signaling pathway. Previous studies [39] showed that SHP-1 activity is upregulated in T cells of aged human donors. Inhibition of SHP-1 in aged T cells in turn increases levels of activated Lck early after activation and augments the cells’ proliferation and functions [39]. These data are in agreement with ours, which show that blockade of BTLA signaling, which augments T cell activation by reducing recruitment of SHP-1, increases T cell responses in half of the aged mice.

Other studies have explored the effects of aging on gene expression profiles of T cells, although none tested subsets of CD8^+^ T cells specific to a single epitope. Cao et al. [26] reported on human peripheral blood derived CD8^+^ T cells from young and aged humans. Similar to our data, they reported defects in CD28 signaling. Other genes that were reported to be differentially expressed in aged T cells such as *Socs3*, a negative regulator of cytokine signaling, *Gfi-1*, a repressor of hematopoiesis or *Gadd45*, induced by DNA damage showed no significant age-related differences in expression in our arrays. Previous studies showed that upon aging, T cells express unusually high levels of receptors that are typically found on natural killer (NK) cells. Levels of transcripts for several NK receptors were significantly different in our comparisons. On A2 CD8^+^ T cells in comparison to those from young AdC68-gDNP induced CD8^+^ T cells, killer cell lectin like receptor (*Klr*) *G1* (FC = −1.9) and *K1* (FC = −1.6) as well as NK cell triggering receptor (*Nktr*, FC = −1.5) differed, but they had lower expression in the aged cells. In comparison to A2 cells, the same transcripts in A1 cells were higher (*Klrg1*, FC = 1.9, *Klrk1*, FC: 1.7, *Nktr*, FC = 1.4), indicating, as had been suggested previously, that expression of these receptors may aid in counter-balancing the effect of immunosenescence on aged CD8^+^ T cells [40].

In summary, our gene expression profiling confirms that TcR/CD28 signaling declines in aged CD8^+^ T cells although this is not linked to reduced expression of the TcR or CD28 on the surface of aged CD8^+^ T cells. The gene expression profile can be restored in half of the aged mice by blockade of the BTLA/CD60 checkpoints during T cell stimulation, indicating increased activities of these immunoinhibitory pathways that contribute to the defects that accumulate in CD8^+^ T cells during immunosenescence.

## METHODS

### Mice

Female 6- to 8-week-old C57Bl/6 mice were purchased from Taconic Labs (Rockville, MD) and kept at the Animal Facility of the Wistar Institute (Philadelphia, PA). All experiments were performed according to institutionally approved protocols. Young mice were tested at >3 months of age. Aged mice were above 18 months of age at the time of vaccination.

### Viruses and vectors

E1-deleted recombinant AdC68 vectors expressing the NP of influenza A virus were generated from a molecular clone. The cDNA encoding the NP gene of the A/PR8 strain under the control of the CMV promoter was inserted into the deleted E1 domain. E1-deleted virus was rescued on HEK 293 cells. The virus was further propagated on HEK 293 cells, purified and titrated as described previously [41]. Vector batches were quality controlled by determining the virus particle to infectious unit ratios, by testing for replication competent adenovirus and for endotoxin contamina-tions. Genetic integrity of the vectors was determined by Southern Blotting of purified viral DNA. Expression of the NP protein was confirmed upon infection of HEK 293 cells by Western Blot analysis.

### Immunization and infection of mice

Mice were immunized i.m. either at 6-12 weeks of age or 18-22 months of age with AdC68NP or AdC68gDNP vector, given i.m. at 10^10^ vp in 100 μl of sterile PBS. For array studies CD8^+^ T cells were purified 14 and 20 days after the injections for the young and aged mice, respectively. For analysis by flow cytometry blood was collected 2 or 4 weeks after vaccination.

### Isolation of lymphocytes

Lymphocytes were isolated from blood and spleen as described previously [10]. Briefly, PBMCs were isolated upon collection of blood via a submandibular bleed into 1ml of 4% sodium citrate and 1ml of RPMI. Lymphocytes were purified by gradient centrifugation with 1ml Histopaque-1083 followed by red blood cell lysis. Splenocytes were isolated upon homogenizing spleens with a screen and plunger, filtration of cells through a nylon screen to remove debris and lysis of red blood cells. Lymphocytes isolated from either compartment were counted under a microscope upon dilution in trypan blue.

### Intracellular cytokine staining and tetramer staining

For tetramer staining purified cells were treated with APC-labeled NP tetramer (NIH Tetramer Core Facility at Emory University, Atlanta, GA) for the immunodominant H-2^b^ class I binding epitope of NP (amino acid sequences 366-374). They were co-stained with a live cell stain, AlexaFlour700-labeled anti-CD44, PerCP-Cy5.5-labeled anti-CD8, and in some experiments PeCy7-labeled anti-CD28. For flow cytometry, at least 300,000 events were collected on a BD LSR II (BD Biosciences, San Jose, CA) and analyzed using FlowJo software.

### Cell sorting

Splenocytes treated with a live cell stain, antibodies to CD8, CD44 and the NP-tetramer were sorted on a BDAria II into live tetramer^+^CD8^+^CD44^+^ cells. On average we obtained 100,000-200,000 cells from young mice while recoveries from aged mice ranged from 15,000 to 135,000 cells per mouse.

### Microarrays

#### RNA isolation, amplification & hybridization

Total RNA and small RNA were isolated from cells using the modified protocol of Ambion RNAqueous-Micro kit. Total RNA was measured by Nanodrop and and by Bioanalyzer using the Agilent RNA 6000 pico kit. Total RNA at 10ng was amplified with the NuGEN Ovation PicoSL WTA system to generate amplified cDNA, which was then labeled with Biotin (NuGEN Encore BiotinIL Module). Biotin labeled cDNA at 750ng obtained from CD8+ T cells of aged and young mice immunized with the AdC68-NP or AdC68-gDNP vaccine was hybridized to Illumina MouseWG-6 v2 whole genome BeadChips. All arrays were processed in the Wistar Institute Genomics Facility.

#### Data preprocessing

Arrays were quantile normalized and the data was filtered to remove non-informative probes that were expressed at background level. Technical replicates available for 9 samples were averaged prior to statistical analysis. All data preprocessing and analysis were performed in MATLAB 7.10.0.

### Statistical analysis

#### Within-class differential gene expression

To assess the changes between the different groups a single-factor ANOVA test (p<0.05) was performed, followed by Tukey's honestly significant difference (HSD), with a 95% confidence interval to control for type I error. The post-hoc test was used to obtain gene lists for four comparisons of interest: aged AdC68-NP versus young AdC68-NP, aged AdC68-gDNP versus young AdC68-gDNP, aged AdC68-gDNP versus aged AdC68-NP, and young AdC68-gDNP versus young AdC68-NP.

An additional ANOVA test was also performed to compare gene expression in A1 and A2 subsets of aged AdC68-gDNP mice with aged AdC68-NP and young AdC68-gDNP mice. Similar criteria were used as described above.

#### Functional analysis

IPA (Ingenuity Systems, www.ingenuity.com) and DAVID [42] were used to identify biological functions and pathways associated with the gene lists generated from the statistical analyses. A pathway or function was deemed significantly enriched if the Benjamini and Hochberg multiple-test corrected p-value was less than 0.05.

## SUPPLEMENTARY MATERIAL FIGURES AND TABLES


