# Supplementary material for: Age-related changes in the gene expression profile of antigen-specific mouse CD8+ T cells can be partially reversed by blockade of the BTLA/CD160 pathways during vaccination
Source: Aging (Albany NY). 2016 Nov 9;8(12):3272–84. doi: 10.18632/aging.101105 (PMC5270668; doi:10.18632/aging.101105)
Supplement: Supplementary file 1 [file aging-08-3272-s001.pdf]

SUPPLEMENTARY MATERIAL

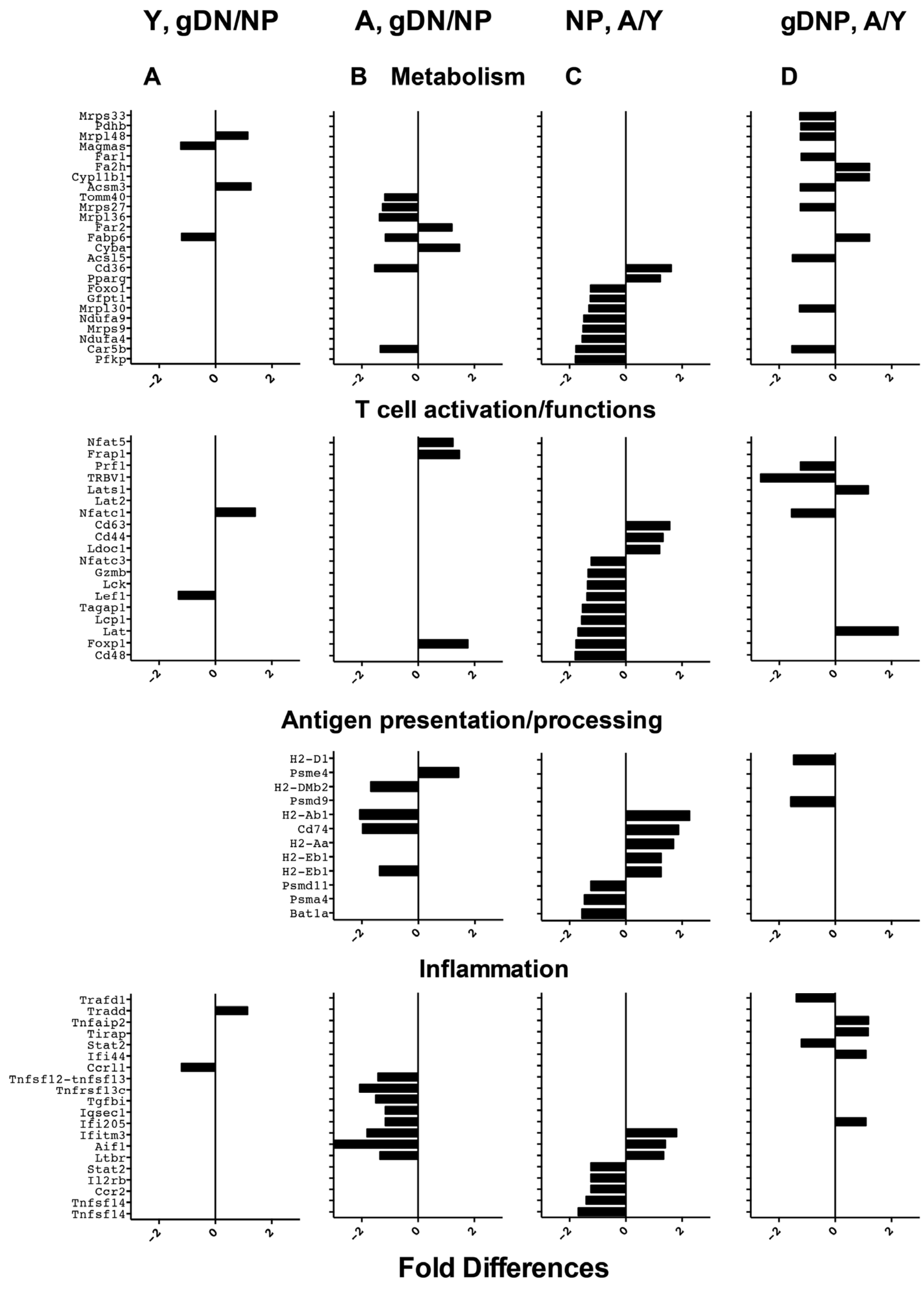

Supplementary Figure 1. The bar graphs show fold differences of the indicated transcripts involved in metabolism and various aspects of immune responses comparing in (**A**) CD8$^+$ T cells from young AdC68-gDNP-vaccinated mice to those of young AdC68-NP-vaccinated mice, in (**B**) CD8$^+$ T cells from aged AdC68-gDNP-vaccinated mice to those of aged AdC68-NP-vaccinated mice in (**C**) AdC68-NP-immune CD8$^+$ T cells from the aged to the younger mice and in **D** AdC68gD-NP-immune CD8$^+$ T cells from the from the aged to the younger mice. Positive values reflect higher expression in younger T cells.

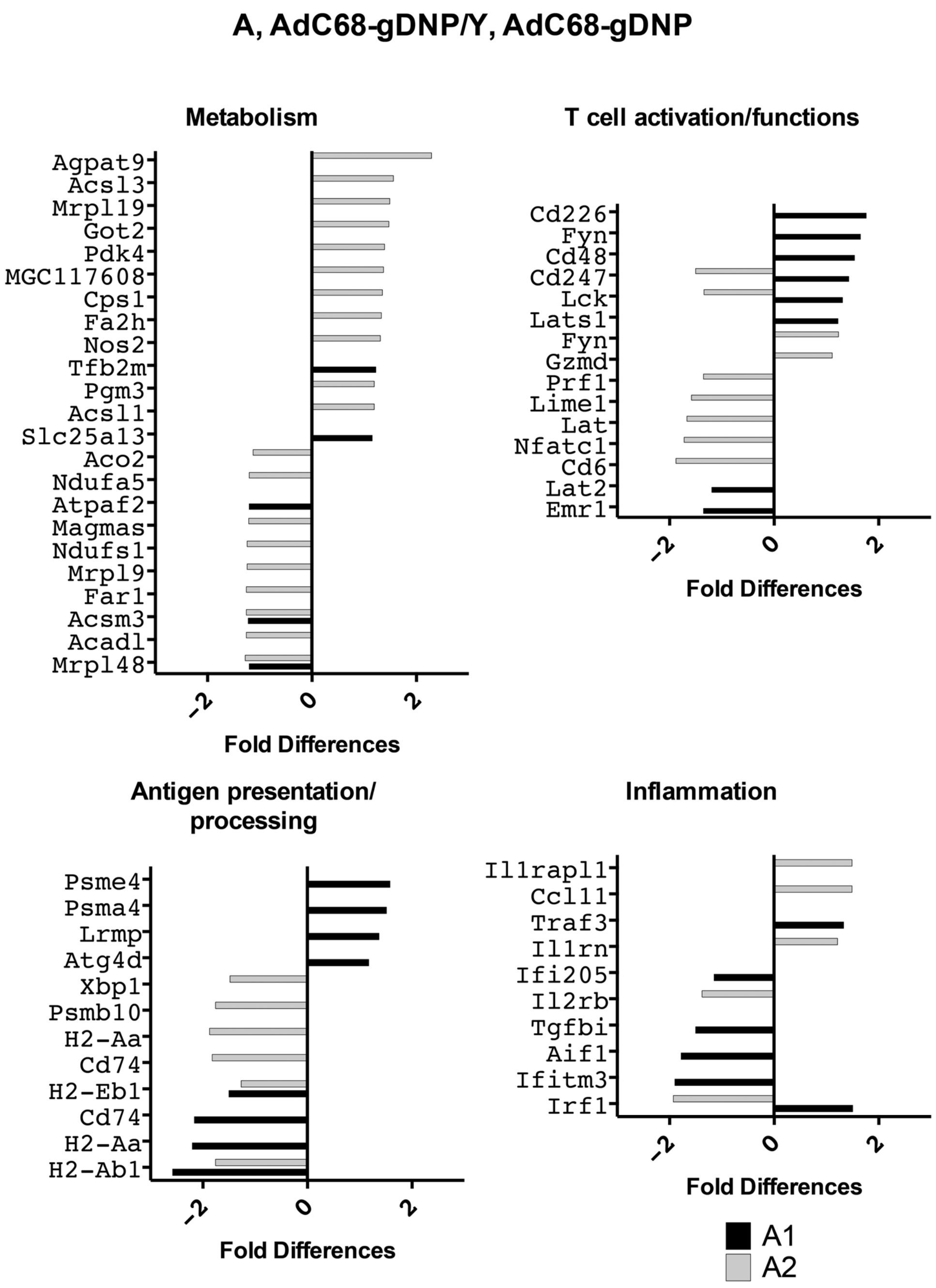

Supplementary Figure 2. The bar graphs show fold differences of the indicated transcripts between the two cohorts of CD8^{+} T cells from aged AdC68-dDNP immunized mice to CD8^{+} T cells from young AdC68-gDNP immunized mice as in Fig 1. Black bars showed fold differences between the A1 group and grey bars reflect data for the A2 group. Positive values reflect higher expression in younger T cells.

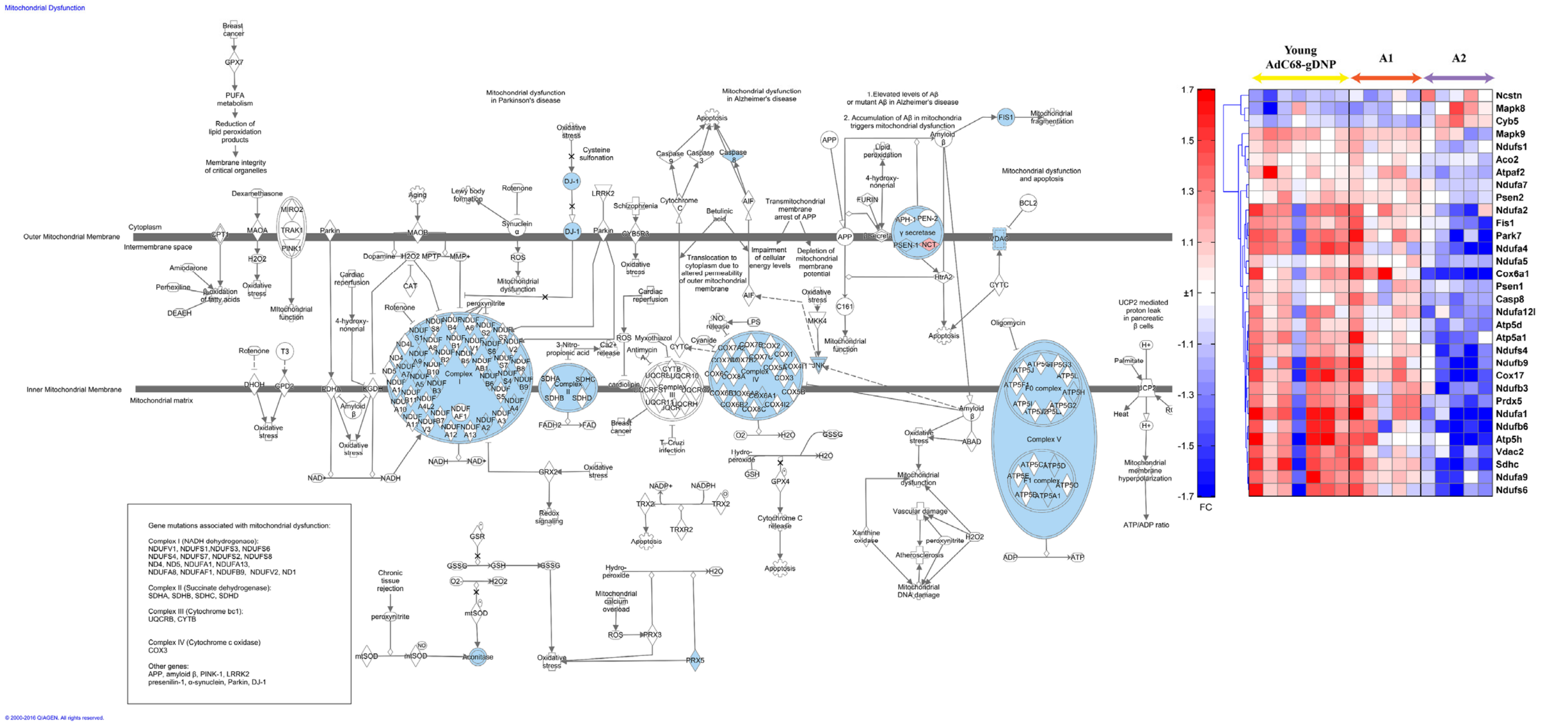

Supplementary Figure 3. Gene expression changes between young AdC68-gDNP and A2 mice in the Mitochondrial Dysfunction pathway from Ingenuity Pathway Analysis. Left: Differentially expressed genes between the two groups are shown (red: upregulated in A2; blue: downregulated in A2). Right: Heatmap of the expression of probes within the pathway in young AdC68-gDNP, A1 and A2 mice.

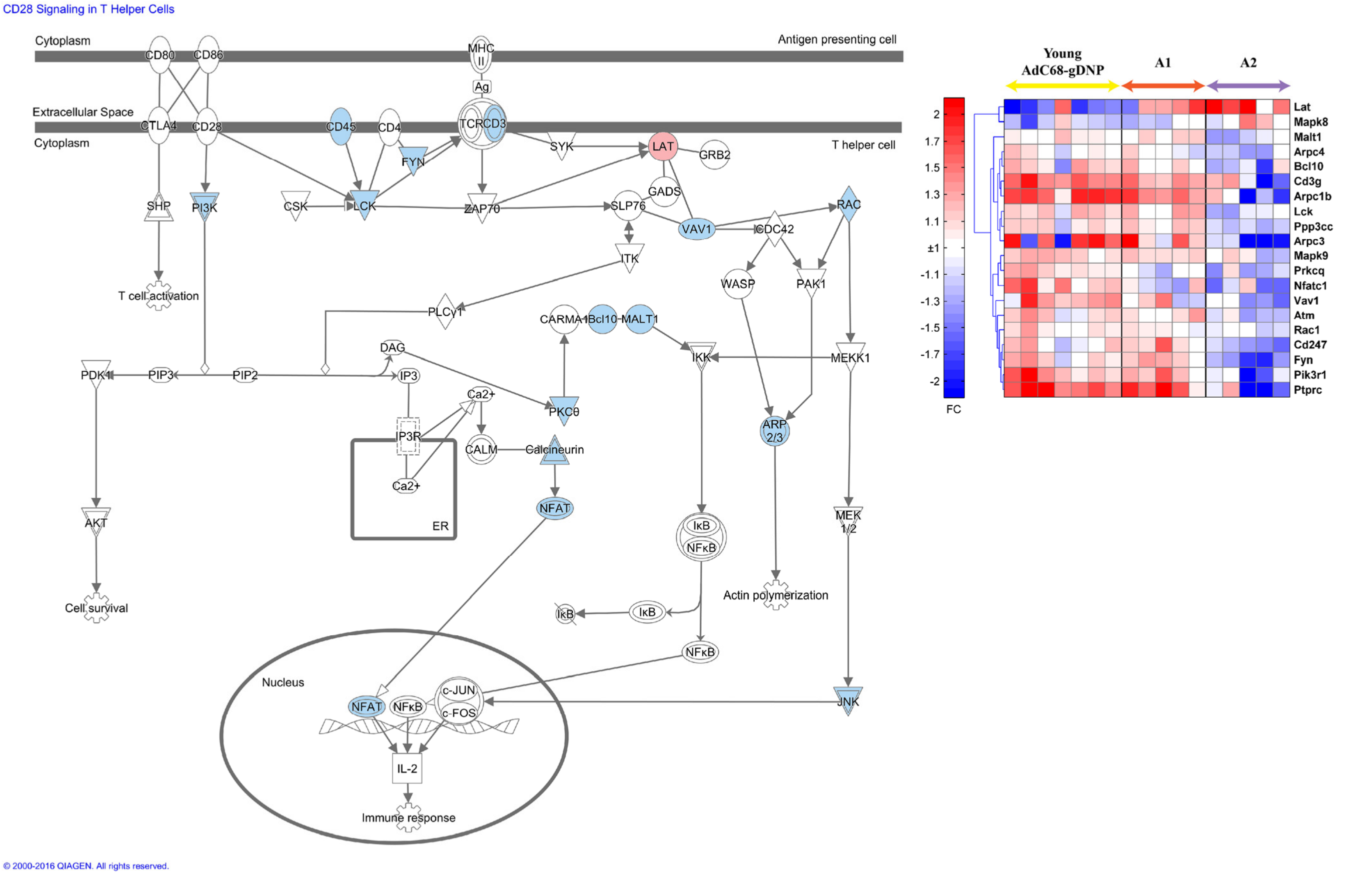

Supplementary Figure 4. Gene expression changes between young AdC68-gDNP and A2 mice in the CD28 Signaling in T helper Cells pathway from Ingenuity Pathway Analysis. Left: Differentially expressed genes between the two groups are shown (red: upregulated in A2; blue: downregulated in A2). Right: Heatmap of the expression of probes within the pathway in young AdC68-gDNP, A1 and A2 mice.

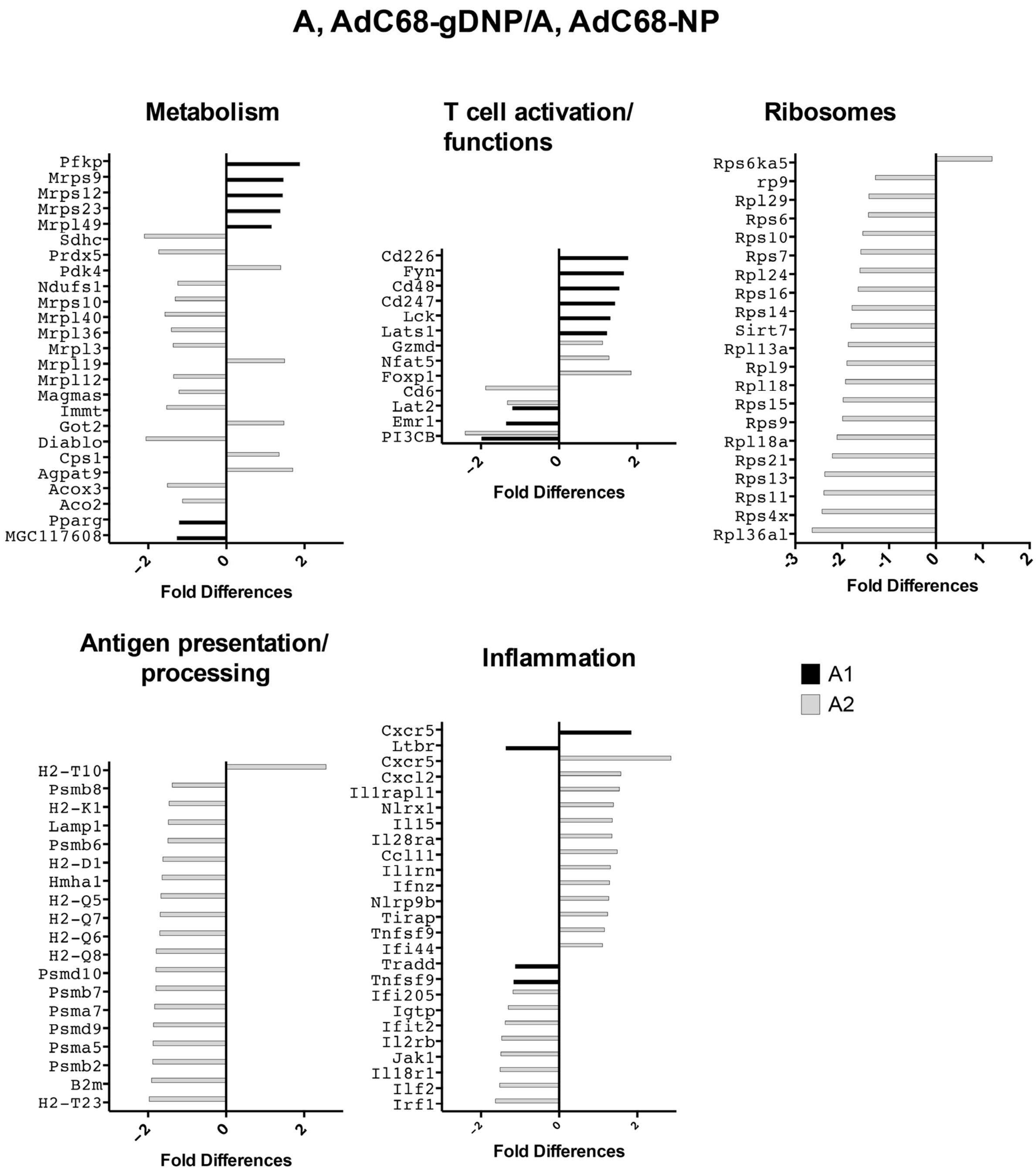

Supplementary Figure 5. The bar graphs show fold differences of the indicated transcripts between CD8[+] T cells from aged AdC68-dDNP immunized mice of groups A1 (in black) and A2 (grey) compared to CD8[+] T cells from aged AdC68-NP immunized mice as in Fig 1. Positive values reflect higher expression in CD8[+] T cells from AdC68-gDNP immunized mice.

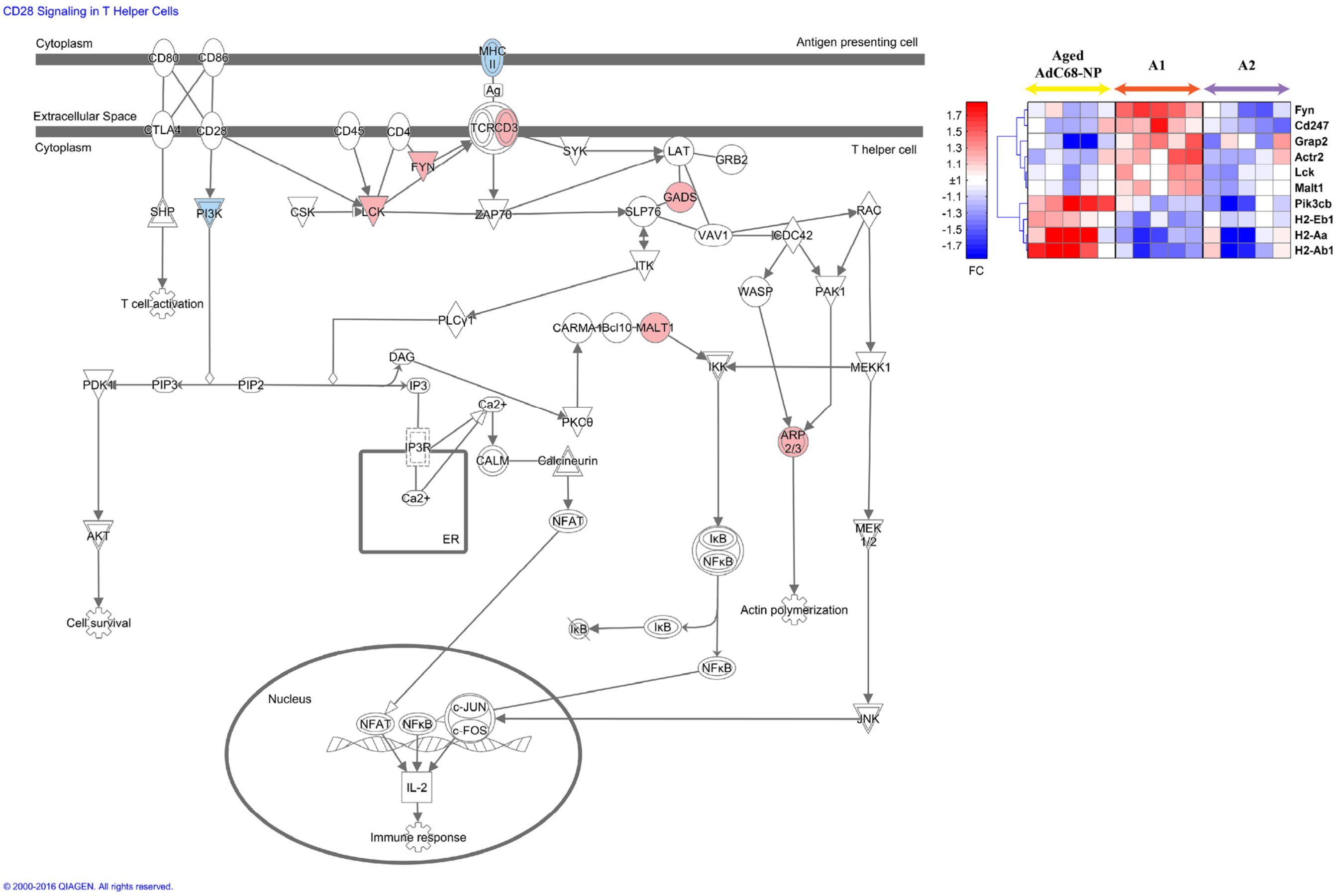

Supplementary Figure 6. Gene expression changes between aged AdC68-NP and A1 mice in the CD28 Signaling in T helper Cells pathway from Ingenuity Pathway Analysis. Left: Differentially expressed genes between the two groups are shown (red: upregulated in A1; blue: downregulated in A1). Right: Heatmap of the expression of probes within the pathway in aged AdC68-NP, A1 and A2 mice.

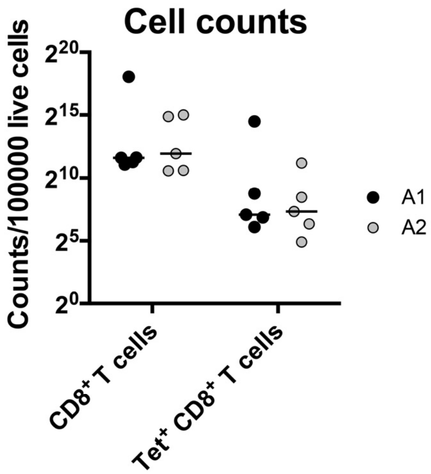

Supplementary Figure 7. The figure shows numbers of CD8$^+$ T cells and NP-tetramer positive CD8+ T cells normalized to 10^5 live lymphoid cells that were analyzed from AdC68-gDNP-immunized mice of the A1 and A2 groups based on results obtained during cell sorting.

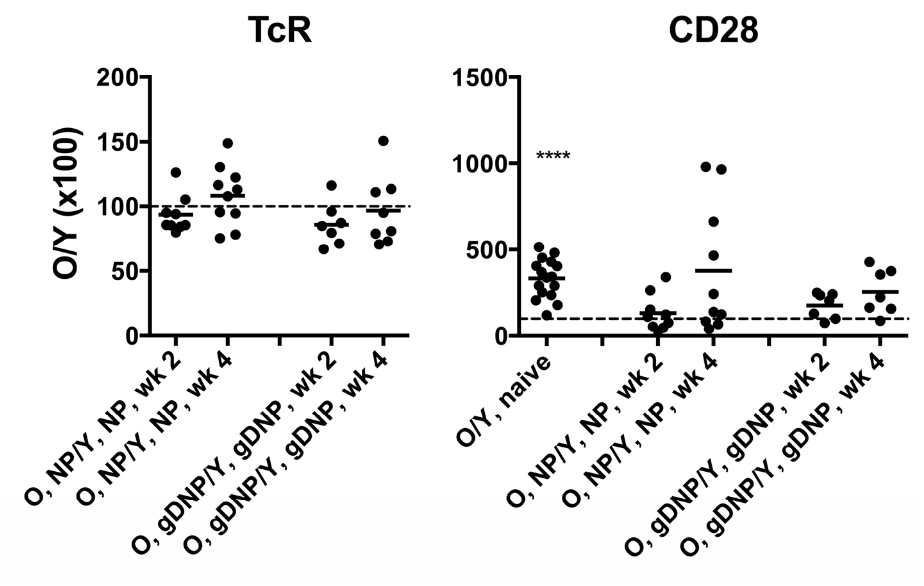

Supplementary Figure 8. The graphs show a comparison for the mean fluorescent intensity (MFI) for staining with the NP tetramer (TcR) and an antibody to CD28 on $CD8^+$ T cells from aged as compared to young mice. Tetramer-positive cells from AdC68-NP (NP)- or AdC68-gDNP (gDNP)-immunized mice were compared at 2 and 4 weeks after immunization. **** indicates a significant difference between the two comparison groups with a p-value <0.0001 (by multiple t-test with type 1 error correction). The dashed line is drawn at 100.

SUPPLEMENTARY RESULTS

Confirmatory studies

We selected 6 transcripts that showed significant differences in expression in the comparison of samples from aged mice immunized with AdC68-NP or AdC68-gDNP and compared levels of their expression by quantitative real time PCR. While the PCR expression data for only 1 of the 6 genes (Sirpa) reached statistical significance, all 6 genes displayed the same direction of fold change and similar magnitude of increase as those of the arrays, as shown in Suppl. Fig. 6

SUPPLEMENTARY METHODS

Analysis by quantitative PCR

Six genes (Klrg1, Lyz, Cd163, Sirpa, Pik3cb, Aif1, Lat2) that were differentially expressed between samples from aged AdC68-NP and aged AdC68-gDNP-immunized mice were selected for analysis using PCR. RNA samples, were isolated using ThermoFisher Scientific RNAqueous®-Micro Kit. 20ng of total RNA was used to generate cDNA using MessageBooster cDNA Synthesis Kit (Epicentre, Manalapan Township, NJ). Quantitative RT-PCR was performed on a Thermo-Fisher (Waltham, MA) Scientific 7900 HT Fast Real-Time PCR System with RT2 SYBR Green Master Mixes, according to the manufacturer. Information for the sequences of the primers from Qiagen (Hilden, Germany) used for the 6 selected genes are listed in Suppl. Table 9. The housekeeping gene Txn1 was used as a loading control. Samples were run in triplicates and 4 replicates where used for each group.

For each gene, triplicates where first averaged, then Ct values were converted to gene expression by calculating the $2{-}\Delta Ct$ value, where ΔCt = Ct gene of interest – Ct of internal control. A t-test was used to compare samples from aged AdC68-gDNP- versus aged AdC68-NP-immunized mice.

Supplementary Table 1. Pathways and functions enriched for genes differentially expressed between young AdC68-NP and young AdC68-gDNP mice

IPA Canonical Pathways	P-Value	Benjamini	# of Genes
SAPK/JNK Signaling	5.89E-04	3.80E-02	4
Wnt/β-catenin Signaling	6.17E-04	3.80E-02	5

Supplementary Table 2. Pathways and functions enriched for genes differentially expressed between aged AdC68-NP and aged AdC68-gDNP mice

IPA Canonical Pathways†	P-Value	Benjamini	# of Genes
Nur77 Signaling in T Lymphocytes	2.29E-05	6.92E-03	7
IL-4 Signaling	1.35E-04	1.70E-02	7
Cytotoxic T Lymphocyte-mediated Apoptosis of Target Cells	1.70E-04	1.70E-02	6
Calcium-induced T Lymphocyte Apoptosis	4.37E-04	3.31E-02	6
Role of NFAT in Regulation of the Immune Response	5.89E-04	3.39E-02	10
PKCθ Signaling in T Lymphocytes	6.76E-04	3.39E-02	8
Graft-versus-Host Disease Signaling	8.51E-04	3.47E-02	5
Role of NFAT in Cardiac Hypertrophy	1.02E-03	3.47E-02	10
Dendritic Cell Maturation	1.15E-03	3.47E-02	9
Amyotrophic Lateral Sclerosis Signaling	1.32E-03	3.47E-02	7

† Top ten significant pathways are listed

Supplementary Table 3. Pathways and functions enriched for genes differentially expressed between young AdC68-NP and aged AdC68-NP mice

IPA Canonical Pathways	P-Value	Benjamini	# of Genes
CD28 Signaling in T Helper Cells	1.45E-05	2.14E-03	11
PKCθ Signaling in T Lymphocytes	1.45E-05	2.14E-03	11
iCOS-iCOSL Signaling in T Helper Cells	2.00E-04	2.00E-02	9
Cytotoxic T Lymphocyte-mediated Apoptosis of Target Cells	3.89E-04	2.40E-02	6
CTLA4 Signaling in Cytotoxic T Lymphocytes	3.98E-04	2.40E-02	8
Calcium-induced T Lymphocyte Apoptosis	9.55E-04	4.79E-02	6
**IPA Biological Functions**	**P-Value**	**Benjamini**	**# of Genes**
Gene Expression	9.72E-07	1.66E-03	67
RNA Post-Transcriptional Modification	4.91E-05	2.10E-02	17
Cell Cycle	1.61E-04	4.42E-02	33
Cancer	3.48E-04	4.42E-02	53
Cellular Development	3.89E-04	4.42E-02	30
Cell Death	4.65E-04	4.95E-02	48

DAVID Functions†	P-Value	Benjamini	# of Genes
SP_PIR_KEYWORDS: phosphoprotein	7.51E-07	2.13E-04	196
GO BP: leukocyte activation	2.26E-04	2.83E-02	16
GO BP: positive regulation of nitrogen compound metabolic process	1.24E-04	2.87E-02	26
GO BP: positive regulation of biosynthetic process	2.22E-04	3.01E-02	26
GO BP: cell activation	1.74E-04	3.14E-02	17
GO BP: positive regulation of transcription	1.17E-04	3.16E-02	24
GO BP: positive regulation of gene expression	1.56E-04	3.17E-02	24
GO BP: positive regulation of transcription from RNA polymerase II promoter	2.20E-04	3.25E-02	19
GO BP: positive regulation of macromolecule biosynthetic process	1.03E-04	3.34E-02	26
GO BP: positive regulation of cellular biosynthetic process	2.11E-04	3.42E-02	26

† Top ten significant functions are listed

Supplementary Table 4. Top 100 differentially expressed probes between young AdC68-NP, young AdC68-gDNP, aged AdC68-NP and aged AdC68-gDNP, ranked according to hierarchical clustering in heatmap (Figure 2).

Illumina ID	Accession	Gene Name	Symbol
ILMN_2806700	NM_007806	cytochrome b-245, alpha polypeptide	Cyba
ILMN_1259185	NM_172991	RIKEN cDNA C030048B08 gene	C030048B08Rik
ILMN_3159185	NM_213616	ATPase, Ca++ transporting, plasma membrane 4	Atp2b4
ILMN_2594066	NM_025620	RIKEN cDNA 2210417D09 gene	2210417D09Rik
ILMN_2648704	NM_198429	nuclear factor of activated T-cells, cytoplasmic, calcineurin-dependent 1	Nfatc1
ILMN_1260262	NM_027098	mitochondrial ribosomal protein L30	Mrpl30
ILMN_1258998	NM_025456	E2F-associated phosphoprotein	Eapp
ILMN_1228498	NM_152822	LAS1-like (S. cerevisiae)	Las1l
ILMN_2615041	NM_054078	bromodomain adjacent to zinc finger domain, 2A	Baz2a
ILMN_2424866	NM_030131	cornichon homolog 4 (Drosophila)	Cnih4
ILMN_2939702	NM_025522	dehydrogenase/reductase (SDR family) member 7	Dhrs7
ILMN_2723907	NM_027213	mediator of RNA polymerase II transcription, subunit 6 homolog (yeast)	Med6
ILMN_2624782	NM_001005509	eukaryotic translation initiation factor 2a	Eif2a
ILMN_3071525	NM_178687	CD226 antigen	Cd226
ILMN_3007669	NM_019693	HLA-B-associated transcript 1A	Bat1a
ILMN_2659528	NM_013836	transcription factor 20	Tcf20
ILMN_1236290	NM_011808	E26 avian leukemia oncogene 1, 5' domain	Ets1
ILMN_2610609	NM_027238	tetratricopeptide repeat domain 39B	Ttc39b
ILMN_1256630	NM_001029889	gene model 608	Gm608
ILMN_3162184	NM_001033201	expressed sequence AU014645	AU014645
ILMN_2444432	AE000663	T cell receptor beta variable 1 207	TRBV1
ILMN_1224128	NM_010902	nuclear factor, erythroid derived 2, like 2	Nfe2l2
ILMN_3065779	NM_027696	mesoderm induction early response 1 homolog (Xenopus laevis	Mier1
ILMN_2837195	NM_026558	RIKEN cDNA 6720467C03 gene	6720467C03Rik
ILMN_2728189	NM_173747	G patch domain and KOW motifs	Gpkow
ILMN_2642969	XR_032069	PREDICTED: similar to Centaurin, beta 1	LOC100045877
ILMN_2677876	NM_138630	Rho GTPase activating protein 4	Arhgap4
ILMN_2462151	NM_199011	diacylglycerol kinase, theta	Dgkq

Illumina ID	Accession	Gene Name	Symbol
ILMN_1215134	NM_198647	TBC1 domain family, member 22B	Tbc1d22b
ILMN_3107690	NM_028527	RIKEN cDNA 1700047I17 gene 1	1700047I17Rik1
ILMN_1225210	AK037664	protein kinase C, theta	Prkcq
ILMN_1225801	AK077734	reticulocalbin 2	Rcn2
ILMN_2794825	NM_153083	thiamine triphosphatase	Thtpa
ILMN_2678547	NM_008866	lysophospholipase 1	Lypla1
ILMN_1260506	NM_011063	phosphoprotein enriched in astrocytes 15A	Pea15a
ILMN_2605268	NM_033526	ubiquilin 4	Ubqln4
ILMN_2548754	AK015797	n/a	4930515K21Rik
ILMN_1236131	NM_198831	mitochondrial ribosomal protein L48	Mrpl48
ILMN_1259355	NM_134033	coiled-coil domain containing 117	Ccdc117
ILMN_2836855	NM_001033251	G protein-coupled receptor 174	Gpr174
ILMN_1227420	XR_031186	PREDICTED: similar to RGD1359202 protein	LOC100045341
ILMN_1232020	XM_618920	PREDICTED: hypothetical LOC544808	LOC544808
ILMN_1228461	XM_110818	n/a	LOC192690
ILMN_2416813	n/a	n/a	4122401K19Rik
ILMN_3015589	NM_008264	homeo box A13	Hoxa13
ILMN_2551741	AK018737	n/a	0610010I05Rik
ILMN_1244257	XM_140912	n/a	LOC227934
ILMN_1231163	XR_032090	PREDICTED: similar to transforming acidic coiled-coil containing protein 1 long	LOC245600
ILMN_1225242	AK077861	n/a	5930426L19Rik
ILMN_1235445	AK084831	nitrogen fixation gene 1 (S. cerevisiae)	Nfs1
ILMN_1251080	XM_354743	similar to fatty acyl elongase	LOC380835
ILMN_2779858	NM_025393	S100 calcium binding protein A14	S100a14
ILMN_3106849	NM_001024717	galactose-3-O-sulfotransferase 3	Gal3st3
ILMN_2900617	NM_053254	transducin-like enhancer of split 6, homolog of Drosophila E(spl)	Tle6
ILMN_2674712	NM_173189	microcephaly, primary autosomal recessive 1	Mcph1
ILMN_2682162	NM_177410	B-cell leukemia/lymphoma 2	Bcl2
ILMN_2674666	XM_987671	PREDICTED: similar to Ig kappa chain V-V region MPC11 precursor	LOC676136
ILMN_1239958	NM_139223	defensin beta 13	Defb13
ILMN_1237656	NM_017395	n/a	Rfx5
ILMN_1238193	AK078731	n/a	7530433C13Rik
ILMN_2668425	NM_007858	diaphanous homolog 1 (Drosophila)	Diap1
ILMN_2877581	NM_172883	major facilitator superfamily domain containing 7	Mfsd7
ILMN_1225565	NM_010512	insulin-like growth factor 1	Igf1
ILMN_1238970	AK081994	n/a	C130092F19Rik
ILMN_1215644	XR_032896	PREDICTED: similar to Eukaryotic translation initiation factor 2 alpha kinase 3	LOC100047634
ILMN_1226525	NM_207105	histocompatibility 2, class II antigen A, beta 1	H2-Ab1
ILMN_2527381	XM_140905	similar to Cathepsin B precursor (Cathepsin B1)	LOC241385
ILMN_1247377	NM_010821	macrophage expressed gene 1	Mpeg1
ILMN_1239102	NM_010382	histocompatibility 2, class II antigen E beta	H2-Eb1
ILMN_2736783	NM_177715	potassium channel tetramerisation domain containing 12	Kctd12
ILMN_2985969	NM_009773	budding uninhibited by benzimidazoles 1 homolog, beta (S. cerevisiae)	Bub1b
ILMN_3112011	NM_130447	dual specificity phosphatase 16	Dusp16
ILMN_2777034	NM_139138	n/a	Emr4
ILMN_2658501	NM_025378	interferon induced transmembrane protein 3	Ifitm3
ILMN_2734729	NM_010378	histocompatibility 2, class II antigen A, alpha	H2-Aa
ILMN_1226525	NM_207105	histocompatibility 2, class II antigen A, beta 1	H2-Ab1

Illumina ID	Accession	Gene Name	Symbol
ILMN_2607675	XM_918601	PREDICTED: similar to MHC class II antigen beta chain	LOC641240
ILMN_2675261	NM_011528	transaldolase 1	Taldo1
ILMN_1246056	NM_001038664	guanine nucleotide binding protein (G protein), gamma transducing activity polypeptide 2	Gngt2
ILMN_1246056	NM_001038664	guanine nucleotide binding protein (G protein), gamma transducing activity polypeptide 2	Gngt2
ILMN_2666007	NM_022325	cathepsin Z	Ctsz
ILMN_2839569	NM_022325	cathepsin Z	Ctsz
ILMN_2834379	NM_009369	transforming growth factor, beta induced	Tgfbi
ILMN_2710382	NM_008172	glutamate receptor, ionotropic, NMDA2D (epsilon 4)	Grin2d
ILMN_1253938	AK019821	homeodomain interacting protein kinase 2	Hipk2
ILMN_1257575	NM_009976	cystatin C	Cst3
ILMN_2722996	NM_007547	signal-regulatory protein alpha	Sirpa
ILMN_1218123	NM_019467	allograft inflammatory factor 1	Aif1
ILMN_1230708	NM_199311	C-type lectin domain family 4, member a1	Clec4a1
ILMN_2738837	NM_022988	Ngg1 interacting factor 3-like 1 (S. pombe)	Nif3l1
ILMN_2781798	NM_001033297	gene model 561	Gm561
ILMN_2680549	NM_029094	phosphatidylinositol 3-kinase, catalytic, beta polypeptide	Pik3cb
ILMN_2729198	BC048004	formin-like 2 (S)	Fmnl2
ILMN_2770270	NM_183286	dehydrogenase/reductase (SDR family) member 13	Dhrs13
ILMN_2826304	NM_008375	fatty acid binding protein 6, ileal (gastrotropin)	Fabp6
ILMN_1221256	NM_152839	immunoglobulin joining chain	Igj
ILMN_2684370	XM_132633	immunoglobulin kappa constant	Igk-C
ILMN_2704562	XM_001476703	PREDICTED: similar to Chain L, Structural Basis Of Antigen Mimicry In A Clinically Relevant Melanoma Antigen System, transcript variant 3	LOC100047628
ILMN_2744657	XM_354710	immunoglobulin heavy constant mu	Igh-6
ILMN_2744660	XM_354710	immunoglobulin heavy constant mu	Igh-6

Supplementary Table 5. Pathways and functions enriched for genes differentially expressed between A1 and young AdC68-gDNP mice

IPA Biological Functions	P-Value	Benjamini	# of Genes
Cell Death and Survival	1.89E-05	2.88E-02	22
Cell Cycle	1.05E-04	4.01E-02	11
Embryonic Development	1.05E-04	4.01E-02	16
Hair and Skin Development and Function	1.05E-04	4.01E-02	3
Renal and Urological System Development and Function	1.05E-04	4.01E-02	4

Supplementary Table 6. Pathways and functions enriched for genes differentially expressed between A2 and young AdC68-gDNP mice

IPA Canonical Pathways†	P-Value	Benjamini	# of Genes
Mitochondrial Dysfunction	1.70E-06	6.03E-04	32
Integrin Signaling	2.40E-06	6.03E-04	36
Caveolar-mediated Endocytosis Signaling	1.74E-05	2.40E-03	17
Paxillin Signaling	1.95E-05	2.40E-03	21
mTOR Signaling	3.09E-05	2.95E-03	31
Molecular Mechanisms of Cancer	3.63E-05	2.95E-03	50
EIF2 Signaling	5.75E-05	4.07E-03	30
Germ Cell-Sertoli Cell Junction Signaling	7.24E-05	4.07E-03	27
Regulation of IL-2 Expression in Activated and Anrgic T Lymphocytes	7.41E-05	4.07E-03	17
Granzyme B Signaling	8.91E-05	4.47E-03	7
**IPA Biological Functions†**	**P-Value**	**Benjamini**	**# of Genes**
RNA Post-Transcriptional Modification	7.75E-13	1.11E-08	69
Cell Death and Survival	2.37E-11	1.69E-07	519
Organismal Survival	9.79E-11	4.66E-07	356
Cellular Growth and Proliferation	2.90E-10	8.31E-07	588
Cancer	3.58E-09	8.53E-06	1347
Gastrointestinal Disease	3.58E-09	8.53E-06	1116
Organismal Injury and Abnormalities	3.58E-09	8.53E-06	1364
Gene Expression	8.75E-09	1.04E-05	342
Cellular Development	1.13E-08	1.23E-05	483
Hematological System Development and Function	1.13E-08	1.23E-05	321
**DAVID KEGG Pathways**	**P-Value**	**Benjamini**	**# of Genes**
KEGG: Ribosome	3.62E-08	6.33E-06	34
KEGG: Dilated cardiomyopathy	6.18E-04	5.27E-02	18
KEGG: Parkinson's disease	8.70E-04	4.95E-02	28
KEGG: Arrhythmogenic right ventricular cardiomyopathy (ARVC)	1.14E-03	4.85E-02	17
**DAVID Functions†**	**P-Value**	**Benjamini**	**# of Genes**
SP_PIR_KEYWORDS: ribonucleoprotein	8.91E-08	4.39E-05	66
SP_PIR_KEYWORDS: acetylation	5.55E-07	1.37E-04	358
GO_CC: ribonucleoprotein complex	1.20E-06	6.11E-04	95
SP_PIR_KEYWORDS: rna-binding	8.26E-06	1.35E-03	91
GO_MF: structural constituent of ribosome	9.04E-06	8.44E-03	39
SP_PIR_KEYWORDS: ribosomal protein	1.16E-05	1.43E-03	44
GO_CC: ribosome	3.15E-05	7.95E-03	46
GO_MF: structural molecule activity	9.27E-05	4.25E-02	62
SP_PIR_KEYWORDS: phosphoprotein	9.83E-05	9.62E-03	743
SP_PIR_KEYWORDS: ribosome	1.16E-04	9.50E-03	12

† Top ten significant pathways/functions are listed

Supplementary Table 7. Pathways and functions enriched for genes differentially expressed between A1 and aged AdC68-NP mice

IPA Canonical Pathways	P-Value	Benjamini	# of Genes
CD28 Signaling in T Helper Cells	7.76E-05	1.35E-02	10
PKCθ Signaling in T Lymphocytes	7.76E-05	1.35E-02	10
**IPA Biological Functions†**	**P-Value**	**Benjamini**	**# of Genes**
Cellular Function and Maintenance	1.44E-08	1.00E-04	129
Hematological System Development and Function	3.11E-08	1.01E-04	102
Cell Morphology	5.27E-08	1.01E-04	91
Cellular Development	1.50E-07	2.08E-04	120
Cellular Growth and Proliferation	1.50E-07	2.08E-04	154
Lymphoid Tissue Structure and Development	3.42E-07	2.77E-04	58
Tissue Morphology	3.44E-07	2.77E-04	77
Organ Morphology	4.94E-07	3.38E-04	71
Hematopoiesis	7.44E-07	3.38E-04	64
Tissue Development	7.44E-07	3.38E-04	96
**DAVID Functions**	**P-Value**	**Benjamini**	**# of Genes**
SP_PIR_KEYWORDS: phosphoprotein	2.07E-05	6.38E-03	202
GO_BP: positive regulation of immune system process	2.78E-05	4.54E-02	17
GO_BP: activation of immune response	2.90E-05	2.39E-02	11
GO_BP: immune response-activating cell surface receptor signaling pathway	5.04E-05	2.76E-02	8
GO_BP: immune response-regulating cell surface receptor signaling pathway	9.47E-05	3.88E-02	8
GO_BP: immune response-activating signal transduction	1.39E-04	4.53E-02	8

† Top ten significant pathways/functions are listed

Supplementary Table 8. Pathways and functions enriched for genes differentially expressed between A2 and aged AdC68-NP mice

IPA Biological Functions†	P-Value	Benjamini	# of Genes
Cellular Development	1.92E-07	1.12E-03	213
Cellular Growth and Proliferation	1.92E-07	1.12E-03	253
Hematological System Development and Function	1.92E-07	1.12E-03	145
Protein Degradation	4.83E-07	1.12E-03	49
Protein Synthesis	4.83E-07	1.12E-03	76
Infectious Diseases	1.32E-06	2.04E-03	107
Cancer	2.09E-06	2.76E-03	495
Organismal Injury and Abnormalities	2.09E-06	2.76E-03	505
Cellular Function and Maintenance	7.67E-06	8.40E-03	154
Cell Death and Survival	9.23E-06	8.54E-03	206

† Top ten significant pathways/functions are listed

Supplementary Table 9. Pathways and functions enriched for genes differentially expressed between A2 and aged AdC68-NP mice

Symbol	Gene Name	Accession	Qiagen catalog #	amplicon size
**Test Genes**				
Rarres1	PREDICTED: retinoic acid receptor responder (tazarotene induced) 1	NM_001164763	PPM28493A	93
Klrg1	killer cell lectin-like receptor subfamily G, member 1	NM_016970	PPM26071A	112
Lyz 1	lysozyme	NM_013590	PPM32410C	131
Cd163	CD163 antigen	NM_053094	PPM06162B	147
Sirpa	signal-regulatory protein alpha	NM_007547	PPM05351E	101
Pik3cb	phosphatidylinositol 3-kinase, catalytic, beta polypeptide	NM_029094	PPM05089A	123
Aif1	allograft inflammatory factor 1	NM_019467	PPM03752A	114
Lat2	linker for activation of T cells family, member 2	NM_022964	PPM59317B	85
**Housekeeping Gene**				
Txn1	thioredoxin 1	NM_011660	PPM35777B	129
